# A descriptive case of persistent *Acanthamoeba* keratitis: raising awareness of this complex ocular disease

**DOI:** 10.1099/acmi.0.000084

**Published:** 2019-11-28

**Authors:** Lisa Connelly, Deepa Anijeet, Claire L. Alexander

**Affiliations:** ^1^​ Scottish Microbiology Reference Laboratories, Glasgow (SMiRL), Glasgow, UK; ^2^​ Ophthalmology, Gartnavel Hospital, Glasgow, UK

**Keywords:** *Acanthamoeba *keratitis, clinical case, molecular testing

## Abstract

**Background:**

Acanthamoeba species are ubiquitous free-living organisms found in the environment. They can cause a sight-threatening disease of the cornea termed *Acanthamoeba* keratitis (AK), often associated with contact-lens wearers. This case review describes a persistent presentation of AK and raises awareness of the challenges faced when diagnosing and managing the disease. It highlights the importance of an accurate and rapid diagnosis to assist patient management and to maximize the potential for a better outcome.

**Case presentation:**

A 73-year-old female was admitted to hospital due to vision impairment of her left eye. Following a clinical examination, the diagnosis of herpes simplex keratitis (HSK) was reported and treated with antivirals. However, deterioration of her keratitis continued after initial treatment, which prompted an investigation into the possibility of AK. Molecular testing of sequential corneal tissue was performed using a real-time PCR assay alongside further clinical examinations. *Acanthamoeba* species DNA was isolated from seven out of eight corneal tissues over a 12 month period. Following prolonged drug treatment and two corneal transplants, the individual’s symptoms ceased and further molecular testing of corneal tissue was negative.

**Conclusions:**

*Acanthamoeba* keratitis can be easily misdiagnosed due to the similarities in the clinical presentation to other, much more common ocular pathogens. This case highlights the importance of considering AK in the first-line diagnosis, and raises awareness that an early, accurate and rapid diagnosis is crucial to improve patient outcome.

## Introduction

*Acanthamoeba* species are isolated from a variety of environmental sources including soil, recreational and drinking water [1]. Although they exist as commensal organisms in the nasal cavity, they can cause serious human infections including AK [2]. This sight-threatening disease of the cornea was first described in 1974 in two patients presenting with chronic eye infections [3]. Common symptoms include ocular pain, irritation, blurred vision, sensitivity to light and excessive tearing. The pain can be severe, deterioration of vision rapid and, if not effectively treated, enucleation and blindness. It has a low incidence (1 in 100 000 in Europe). These symptoms can last for months and, depending on the severity of the infection, can lead to partial or total sight loss.

*Acanthamoeba* keratitis is commonly associated with contact-lens use but can also be reported from non-contact-lens wearers. About 85 % of cases of AK occur in contact-lens wearers, due to improper lens wear, although the disease may occur after corneal trauma, especially in rural settings. Associated risk factors include rinsing lens with contaminated tap water or swimming whilst wearing contact lenses [1]. According to the Association of Contact Lens Manufacturers (ACLM) in 2017, there were approximately 4.2 million contact-lens wearers in the UK. To date, there are 24 *Acanthamoeba* species and 20 genotypes termed T1 to T20. The most common genotype identified from cultured isolates is T4, which has been shown to be related to keratitis infections [4].

Laboratory techniques for AK diagnosis in ocular samples are based on a combination of clinical presentation and laboratory identification. Laboratories across the UK largely culture Acanthamoeba from corneal tissue (commonly referred to as corneal scrapes). This involves the use of non-nutrient agar plates seeded with *Escherichia coli *. If *Acanthamoeba* species are present, feeding tracks are observed in the media. This technique has its limitations as it is insensitive, time consuming and may take several weeks of prolonged incubation to obtain a result. In addition to culturing, direct microscopy of cysts and trophozyotes can be performed using a variety of stains to enhance their appearance [5]. Confocal microscopy is also a useful tool to aid diagnosis but requires advance training, expertise and is only available at specialist centres. In recent years, advances in laboratory techniques to aid diagnosis have resulted in improvements. These include the implementation of molecular tests such as conventional PCR assays and real-time PCR assays [6], [7] and [8]. These sensitive assays permit a rapid diagnosis with results being obtained within 3 h.

This case report describes a challenging, severe case of AK to raise awareness of this sight-threating disease and to highlight the importance of an accurate, rapid diagnosis to minimize ocular deterioration.

## Case report

A 73-year-old female, who wore disposable soft contact lenses presented to a Canadian Eye Department. Two weeks prior to admission, the individual began to experience irritation in her left eye and diminished sight, which were not resolving. On examination, she received a presumptive diagnosis of HSK. She was treated with combination therapy comprising of Zovirax eye ointment (an anti-viral drug with the active ingredient Aciclovir) and topical antibiotics.

Immediately on return to the UK, the patient attended their local Scottish National Health Service (NHS) Eye Department. Clinical symptoms consisted of photophobia, redness and intense pain in the left eye. A 3 week treatment course was given, including a continuation of Zovirax eye ointment along with antibacterial and topical steroids. Symptoms worsened with the patient describing rapid deterioration of vision combined with severe left-eye pain.

**Fig. 1.**
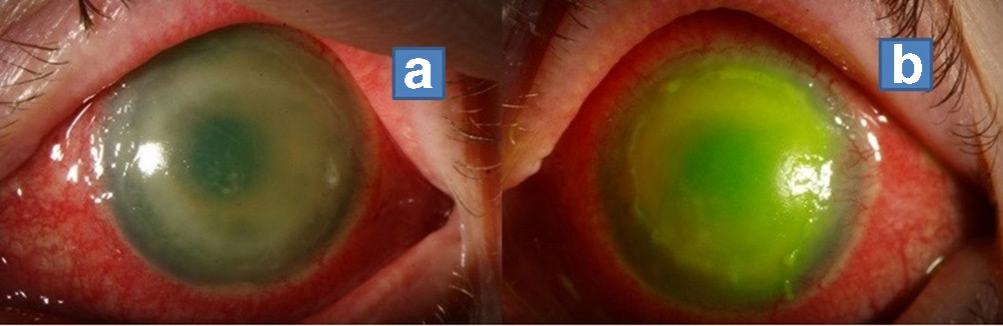
Different stages of *Acanthamoeba* keratitis in a patient. (a) *Acanthamoeba* stromal ring infiltrate at patient’s admission. (b) With florescein stain showing no improvement after 6 weeks with anti-*acanthamoeba* treatment.

The patient was then referred to a specialist Scottish Ophthalmology centre where sight deterioration continued with visual acuity described as hand movements, and able to count fingers. Following a slit-lamp examination, a ring infiltrate of the cornea was visible in the patient’s left eye. On performing the slit-lamp examination, a typical appearance of an Acanthamoeba ring-like form was observed (Fig. 1). Treatment for AK was started on a clinical basis, which comprised of the combination therapy, biguanide and diamidine. Corneal tissue derived from scraping the surface of the eye following the addition of fluorescein was removed, placed into transport media and sent directly to the Scottish Microbiology Reference Laboratories (SMiRL), for molecular testing.

On arrival at the laboratory, the sample was subjected to automated DNA extraction (easyMAG, BioMérieux) and molecular amplification using a real-time PCR assay. The DNA from each sample was ampliﬁed in duplicate as described by Alexander *et al*. [6]. Ampliﬁcation of the human albumin gene was also included as an internal control [9] (Table 1). The cycling conditions consisted of 95 °C for 10 min, followed by 40 cycles of 95 °C for 15 s, 60 °C for 60 s and 40 °C for 5 s in a 25 µl reaction volume. A positive control (*A. castellanii*, reference 1501/1A) and a negative control, with no template and consisting of transport medium were run in each assay.

**Table 1.** Sequences of Acanthamoeba-speciﬁc and internal control oligonucleotides

Gene	Oligonucleotide	Sequence (5′→3′)	References
18S rDNA	Acanthamoeba forward primer (TaqAcF1)	CGACCAGCGATTAGGAGACG	[7]
	Acanthamoeba reverse primer (TaqAcR1)	CCGACGCCAAGGACGAC	
	Acanthamoeba probe (TaqAcP1)	FAM-TGAATACAAAACACCACCATCGGCGC-BHQ2	
human albumin	Albumin forward primer	TGAAACATACGTTCCCAAAGAGTTT	[9]
	Albumin reverse primer	CTCTCCTTCTCAGAAAGTGTGCATAT	
	Albumin probe	VIC-TGCTGAAACATTCACCTT CCATGCAGA-TAMRA	

The presence of *Acanthamoeba* species DNA was reported by molecular testing, providing supportive laboratory evidence for an AK diagnosis. Following 4 weeks of treatment, a further corneal tissue was submitted for molecular testing, which was also shown to be positive. On a monthly basis, a total of four follow-up corneal tissues were submitted and all four samples were reported to contain Acanthamoeba DNA.

**Fig. 2.**
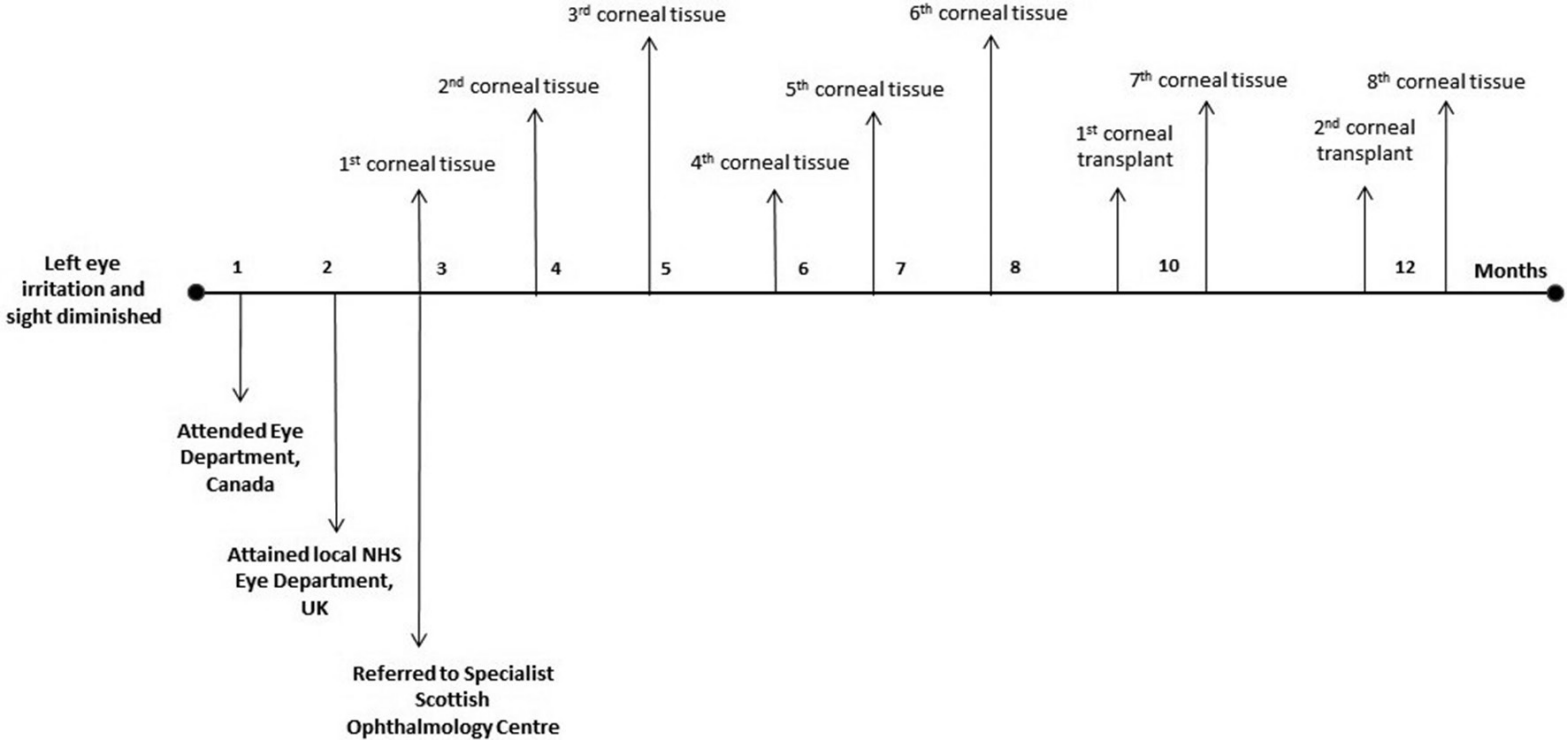
Timeline highlighting case patient diagnosis.

Despite continued topical treatment, the keratitis failed to resolve, with molecular evidence of persistent Acanthamoeba activity. Following unsuccessful topical treatment, a corneal transplant was performed 8 months after the initial diagnosis. This first transplant was unsuccessful due to tissue dehiscence possibly due to persistent Acanthamoeba infection. A positive Acanthamoeba PCR finding was noted 19 days post-transplant. A second transplant was performed 10 weeks later. Further molecular testing was performed 11 days later after this second transplant and was found to be negative. No clinical recurrence was observed and the second transplant was deemed successful. The timeline of prognosis to being treated is highlighted in Fig. 2.

## Discussion

This report highlights that diagnosing and managing AK can be extremely challenging. Risks associated with AK include poor hygiene especially when handling and cleaning contact lenses, and wearing contact lens when bathing or swimming [1]. In this case, the individual expressed good hygiene practice when handing both contact lens and the contact-lens case. However, it was revealed that she had been wearing her contact lens whilst swimming a few months before her first admission to the Canadian Eye Department, which may have been a contributing factor.

The challenges in managing AK cases are confounded by the ability of Acanthamoeba to mimic other ocular pathogens including viruses, bacteria and fungi [10]. Although Zovirax is the recommended treatment for HSK, it is not uncommon for it to be given inappropriately to AK individuals when mis-diagnosis occurs. This delay in giving the correct treatment is likely to result in a poorer outcome [1]. The treatment regime for AK needs to be administered promptly to minimize aggressive disease and often has to be given over lengthy periods. In this reported case, the patient was advised to administer the combination therapy of biguanide and diamidine every hour.

In addition to a rapid diagnosis, improved treatment approaches are required for challenging AK cases to minimize the need for surgical intervention. At present, there are no health-service laboratories in the UK that offer Acanthamoeba drug-resistance testing. Furthermore, corticosteroid therapy may lead to delayed anti-amoebic treatment, as well as stimulating the proliferation of Acanthamoeba, therefore, worsening the prognosis [11]. In this particular case, the patient was treated with corticosteroids, which may have contributed toward disease progression. This is supported by a recent case review, which highlighted the misdiagnosis of AK as HSK [12]. This emphasizes the importance of a correct diagnosis to prevent unnecessary treatment being administered.

Of note is that the Acanthamoeba ring-like infiltrates, which are a helpful clinical feature of AK, were not identified until the individual’s third hospital visit. This observation, combined with molecular testing was crucial to support the diagnosis. In 2015, Alexander *et al.* introduced the first national service in Scotland for the rapid and sensitive molecular identification of *Acanthamoeba* species directly from clinical samples, thus avoiding the need for culture.

**Fig. 3.**
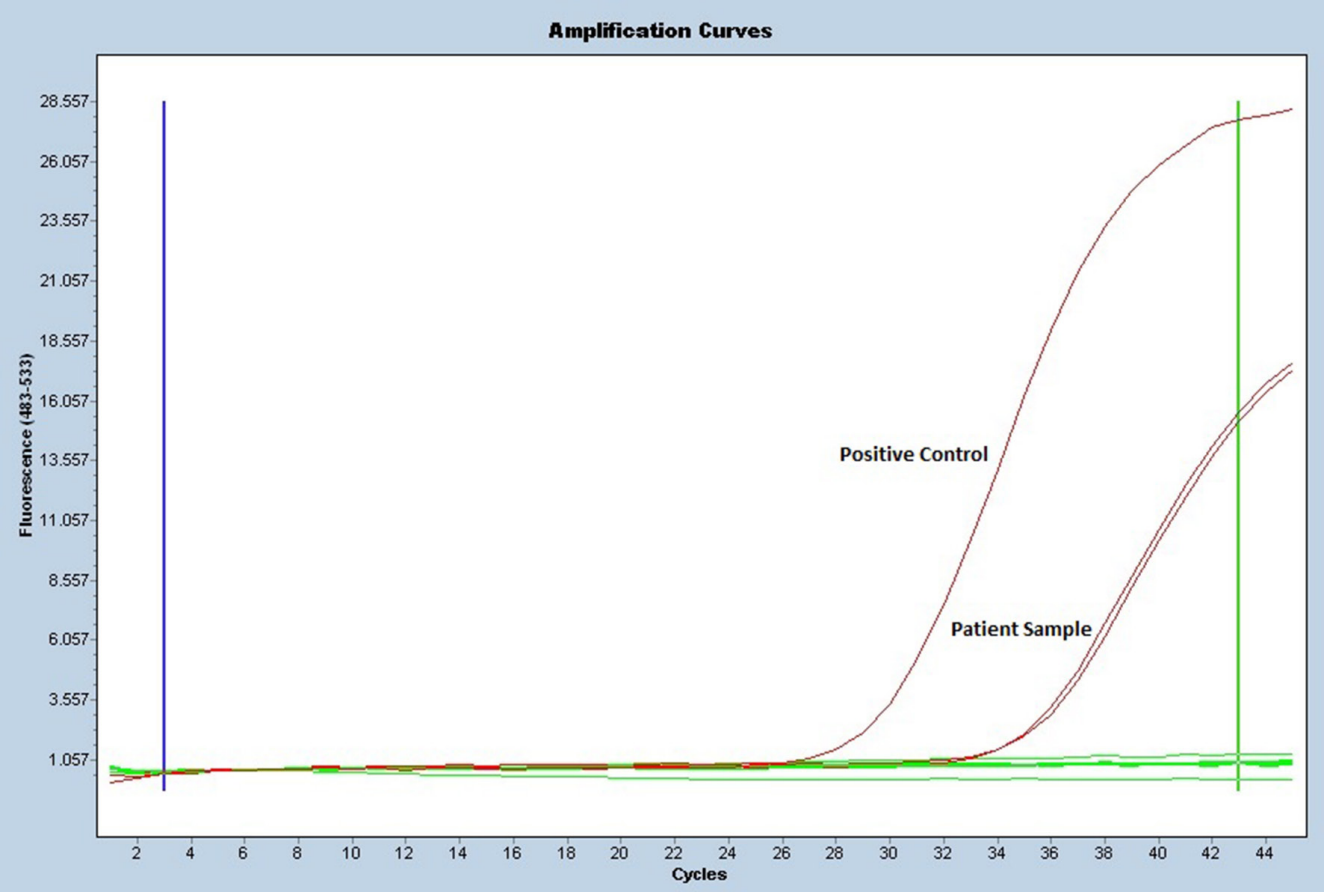
Diagnostic and reference parasitology service *Acanthamoeba* real-time PCR positive traces.

Since implementing this service in 2014 there have been 164 Acanthamoeba real-time PCR positives. That consist of 36 in 2014 (107 tested), 25 in 2015 (278), 28 in 2016 (353), 40 in 2017 (388), 20 in 2018 (326) and so far, 15 in 2019 (229). These figures highlight the importance of the continuation of this service for the detection of this opportunistic pathogen on ocular samples throughout the UK, including this case patient. Typical positive real-time PCR traces obtained from ocular samples are shown in Fig. 3. This case highlights the importance of collaboration between both the clinical laboratory services and the ophthalmology experts to ensure appropriate patient management.

Further work is on-going to investigate the in-depth molecular profiling of the Acanthamoeba DNA from this case to explore the possibility that certain genotypes confer more aggressive disease. As reports of AK within the UK have increased over the past 10 years [13], it is important to gain a better understanding of this potentially sight-threatening disease. Raising awareness ensures clinicians consider Acanthamoeba alongside other ocular pathogens and highlights the benefits of molecular testing to encourage other laboratories to consider a similar approach. By achieving a rapid diagnosis, long-term consequences requiring surgical intervention to prevent partial or total sight loss can be avoided.
